# Impact of the COVID-19 pandemic on outpatient follow-up among people living with HIV retained in care at a University Center in Brazil

**DOI:** 10.1016/j.clinsp.2026.100966

**Published:** 2026-04-23

**Authors:** Mariana Velho Menoia, Guilherme Barbosa Pinto, Erika Yukie Ishigaki, Nicoly Caroline de Andrade Delmondes, Daniel Ayabe Ninomiya, Olavo Henrique Munhoz Leite, Marcello Mihailenko Chaves Magri

**Affiliations:** a Discipline of Infectology, Centro Universitário FMABC, Santo André, SP, Brazil; b Division of Clinic of Infectious and Parasitic Diseases, Hospital das Clínicas, Faculdade de Medicina, Universidade de São Paulo (HCFMUSP), São Paulo, SP, Brazil

**Keywords:** COVID-19, HIV, Outpatient care, Pandemic, Brazil, Continuity of care

## Abstract

**Objective:**

To evaluate the impact of the COVID-19 pandemic on outpatient follow-up and clinical outcomes of People Living with HIV (PLHIV) at a specialized infectious diseases clinic in southeastern Brazil.

**Methods:**

A retrospective analysis was conducted using medical records and responses to a structured questionnaire from PLHIV followed between March 2020 and December 2021. Clinical and laboratory data (CD4 count, viral load, and antiretroviral therapy changes) were reviewed, and questionnaire responses were used to assess COVID-19 history, vaccination, and mental health symptoms.

**Results:**

A total of 125 patients were included (mean age 38.6-years; 64.1% male). Prior to the pandemic, 80.3% had CD4 counts > 200 cells/µL and 82.1% had undetectable viral load, increasing to 89.7% and 84.6%, respectively, during the pandemic. Twenty-six patients (22.2%) changed their antiretroviral regimen, mainly due to switching to the nationally recommended first-line therapy. Among the 55 respondents to the questionnaire, 13 (24%) reported confirmed COVID-19 infection; of these, 2 (15.4%) required hospitalization. Overall, 53 (96.4%) reported having received the COVID-19 vaccination. Symptoms of depression and anxiety were reported by 27.7% and 45.4% of participants, respectively.

**Conclusion:**

Despite the logistic challenges associated with the COVID-19 pandemic, viral suppression, immunologic recovery, and retention in care remained stable in this selected cohort of patients with continuous follow-up, reflecting the resilience of the healthcare service and sustained treatment adherence.

## Highlights

• HIV care remained stable in patients retained during COVID-19.
• Viral suppression and CD4 counts were maintained over time.
• Telemedicine and ART dispensing were used during the pandemic.
• Anxiety and depressive symptoms were frequently reported.
• Findings reflect outcomes in a selected cohort with follow-up.

## Introduction

After the declaration of the COVID-19 pandemic in March 2020, the Brazilian healthcare system underwent substantial structural and logistic changes that affected the continuity of care for patients with chronic diseases. Confinement measures designed to curb transmission also increased the risk of anxiety, depression, other psychological disorders and a functional decline.^1^, ^2^, ^3^

Among those requiring regular outpatient follow-up, People Living with HIV (PLHIV) represent a particularly vulnerable population. According to UNAIDS, in 2020, there were 37.7 million people living with HIV worldwide, with 1.5 million new infections and 680,000 AIDS-related deaths. About 27.5 million individuals were receiving antiretroviral therapy (73% of all PLHIV). Globally, 84% of PLHIV knew their status, 73% were on ART, and 66% achieved viral suppression.^4^ Latin-American studies have reported loss-to-follow-up rates between 8% and 30%.^5^ a situation likely worsened by pandemic restrictions.^5^^,^^6^

The indirect effects of COVID-19 on HIV care are therefore a concern, as many in-person consultations were canceled or replaced by remote visits. International agencies such as the CDC and WHO urged countries to maintain uninterrupted HIV services and safeguard the rights of PLHIV.^7^ In response, several countries adapted service delivery; Thailand and the United States extended ART dispensing intervals to 3–6 months.^8^ In Brazil, tele-prescriptions, online counseling on PrEP and PEP, and partnerships with NGOs facilitated medication access.^9^ This study evaluated whether such disruptions negatively affected outpatient follow-up of PLHIV at a specialized infectious-disease center in southeastern Brazil, aiming to provide evidence to improve post-pandemic strategies for HIV care.

## Methods

This was a retrospective, descriptive, and analytical observational study carried out at the outpatient clinic for infectious and parasitic diseases of Centro Universitário FMABC, in Santo André, São Paulo State, southeastern region of Brazil. The clinic is a referral center within the Brazilian Unified Healthcare System (SUS) that provides specialized multidisciplinary care for People Living with HIV (PLHIV). The objective was to evaluate the impact of the COVID-19 pandemic on the continuity of outpatient follow-up between March 2020 and December 2021, comparing data obtained during this period with those from the year 2019, immediately preceding the pandemic.

**Fig. 1:**
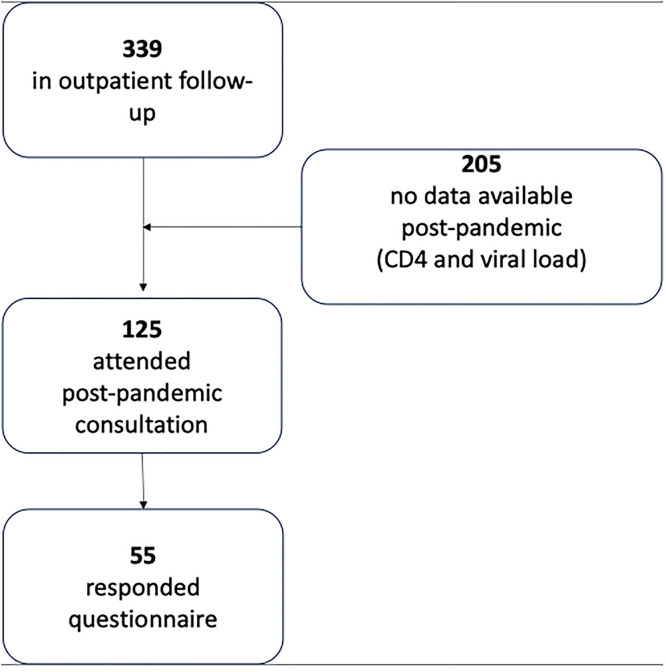
Overall study flowchart.

All adults aged 18-years or older with a confirmed HIV diagnosis and regular follow-up at the clinic before March 2020 were eligible. Inclusion required at least one clinical visit and available laboratory data, CD4 T-cell count, and plasma viral load, both before and during the pandemic, as well as a recorded Antiretroviral Therapy (ART) prescription. Patients with incomplete records or missing paired laboratory results were excluded from the analysis (Fig. 1).

Data were collected from electronic and paper medical charts using a standardized form developed by the investigators. Demographic information (age, sex, education, and occupation), date of HIV diagnosis, duration of follow-up, current ART regimen, and any modifications during the study period were recorded. To complement clinical data, a structured questionnaire was applied between August 2021 and February 2022, either in person or electronically through a secure online platform. The questionnaire explored self-reported adherence to ART, access to appointments and medications during the pandemic, history of COVID-19 infection and vaccination, and perception of mental-health status.

Mental health was screened using two validated instruments: The Patient Health Questionnaire-2 (PHQ-2) for depressive symptoms and the Generalized Anxiety Disorder Scale-2 (GAD-2) for anxiety. Each consists of two questions scored from 0 (“not at all”) to 3 (“nearly every day”); a total score ≥3 indicated a positive screen for the respective condition. These brief scales were selected for their practicality in routine outpatient and telemedicine assessments.

Clinical and laboratory parameters followed national and international definitions. Viral suppression was defined as plasma HIV RNA 〈 50 copies/mL, and immunological recovery as a CD4 T-cell count 〉 200 cells/µL. Changes in ART were categorized according to the reason documented in the record: switch to the nationally preferred first-line regimen (tenofovir + lamivudine + dolutegravir), adverse drug reactions or intolerance, virological failure, patient preference, or drug unavailability. Information on confirmed COVID-19 diagnosis, need for hospitalization, and vaccination status, including number and type of doses received, was also obtained.

During the study period, the outpatient clinic adopted emergency measures to reorganize care delivery, including the use of telemedicine and extended medication dispensing, as part of broader efforts to reorganize services and reduce potential risks to patients. These measures were implemented as emergency organizational adaptations and were not systematically documented at the individual patient level.

All data were anonymized and entered into a protected database. Quantitative variables were expressed as means ± standard deviation or as medians and interquartile ranges, depending on distribution, and categorical variables as absolute numbers and percentages. Comparisons between pre-pandemic and pandemic data were performed with paired statistical tests: Student’s *t*-test or Wilcoxon signed-rank test for continuous variables and McNemar or χ^2^ tests for categorical variables. A two-tailed p-value < 0.05 was considered statistically significant. Analyses were performed using IBM SPSS Statistics version 26.0 (IBM Corp., Armonk, NY, USA). The study was conducted and reported in accordance with the STROBE Statement for observational studies.

The study was approved by the Research Ethics Committee of Centro Universitário FMABC (CAAE 59844222.0.0000.0082). All participants provided written informed consent before inclusion, and confidentiality was preserved in accordance with the Brazilian General Data Protection Law (LGPD, Law 13.709/2018) and the principles of the Declaration of Helsinki.

## Results

A total of 125-patients met the inclusion criteria and were analyzed. The mean age was 48±12 years, and 64.0% were male. Most participants had completed high school (64.0%). The median duration of HIV infection was 8-years (IQR 4–13). The main sociodemographic and laboratory characteristics of the cohort are presented in Table 1, Table 2.

**Table 1:** Sociodemographic and clinical characteristics of people living with HIV followed at a specialized outpatient center in southeastern Brazil.

Variable	Category	n = 125	%
Sex	Male	80	64.0
Race/Skin color	White	47	37.6
	Brown (Parda)	17	13.6
	Black	20	16.0
	Not recorded	40	32.0
Degree of Schooling	< 10-years	22	17.6
	10–12 years	44	35.2
	Some college or higher	36	28.8
	Not recorded	23	18.4
HIV transmission route	Vertical	3	24.0
	Sexual	72	57.6
	Bloodborne	8	6.4
	Not recorded	42	37.0
Comorbidities	None	62	49.6
	Hypertension	14	11.2
	Dyslipidemia	12	9.6
	Hepatitis C	11	8.8
	Diabetes mellitus	9	7.2
	Depression	9	7.2
	Fractures/Osteopenia	5	4.0
	Hepatitis B	2	1.6
	Chronic lung disease	1	0.8

**Table 2:** Laboratory parameters of people living with HIV before and during the COVID-19 pandemic.

	Pre-pandemic	Pandemic	95% IC	p-value
**CD4 (cells/µL), mean ± SD**	541 ± 371	545 ± 336	−43.9 ‒ 36.3	0.852
CD4 < 100, n (%)	12 (9.8)	7 (5.7)		
CD4 100‒200, n (%)	13 (10.6)	13 (10.6)		
CD4 > 200, n (%)	98 (79.7)	103 (83.7)		
**Viral load (copies/mL), mean ± SD**	18,391 ± 78,403	192 ± 1167	4313.9 ‒ 32,083-5	0.011
< 50 copies	97 (77.6)	110 (88.7)		
50‒1000 copies	14 (11.2)	9 (7.3)		
> 1000 copies	14 (11.2)	5 (4.0)		
Undetectable viral load^a^, n (%)	91 (72.8)	109 (87.2)		

a < 50 copies/mL.

Before the COVID-19 pandemic, the mean CD4 T-cell count was 541 cells/µL, and 72.8% of patients had an undetectable viral load (< 50 copies/mL). During the pandemic period, the mean CD4 T-cell count was 545 cells/µL, and 87.2% of patients had suppressed viral load. The proportion of patients with virological suppression below the predefined threshold did not differ significantly between the pre-pandemic and pandemic periods. However, when viral load was analyzed as a continuous variable using paired pre-post comparisons, a statistically significant reduction in viral load levels over time was observed (p = 0.011).

A structured questionnaire was completed by 55-patients (44.0% of the cohort). Among these respondents, 33 (60.0%) reported having undergone COVID-19 diagnostic testing, of whom 13 (39.4%) had positive results, corresponding to 24% of the total respondents. Among those with confirmed infection, 2 (15.4%) required hospitalization, both with favorable recovery. Table 3, Table 4 present the main findings from the COVID-19 ‒ related questionnaire, including symptoms, preventive measures, and the perceived effects of the pandemic on HIV care.

**Table 3:** Responses to the COVID-19 ‒ related questionnaire among people living with HIV (n = 55).

Section	Question / Symptom	n (%)
Flu-like symptoms	Reported flu-like symptoms	38 (69.1)
	Cough	29 (52.7)
	Headache	29 (52.7)
	Runny nose	25 (45.5)
	Fever	20 (36.4)
	Sore throat	18 (32.7)
	Myalgia	17 (30.9)
	Dyspnoea	9 (16.4)
	Loss of taste	5 (9.1)
	Anosmia	4 (7.3)
	Diarrhea	4 (7.3)
	Generalized weakness	3 (5.5)
	Nausea	3 (5.5)
Duration of symptoms	Less than a week	22 (40.0)
	More than a week	12 (21.8)
	Two or more weeks	4 (7.2)
COVID-19 testing	Tested for COVID-19	33 (60.0)
	Positive tests (among tested)	13 (39.4)
	Hospitalization due to COVID-19	2 (15.4)
	Need for ventilatory support (among hospitalized)	2 (100)
Mental health screening	PHQ-2 ≥ 3 (depressive symptoms)	15 (27.7)
	GAD-2 ≥ 3 (anxiety symptoms)	28 (45.4)
Perception of COVID-19 risk	Very worried about being infected	10 (18.5)
	A little worried	32 (59.3)
	Not worried	12 (22.2)
	Very worried about infecting a loved one	23 (42.6)
	A little worried	23 (42.6)
	Not worried	8 (14.8)
Knowledge of preventive measures	Feels informed	49 (89.1)
Protective measures adopted	Frequent handwashing	33 (60.0)
	Hand sanitizer use	33 (60.0)
	Mask use	30 (54.5)
	Covering mouth when coughing/sneezing	27 (49.1)
	Handwashing after coughing/sneezing	24 (43.6)
	Avoid touching face	17 (30.9)
	Social distancing	15 (27.3)
	Body temperature monitoring	1 (1.8)
COVID-19 vaccination	Vaccinated	53 (96.4)
	Two doses	3 (5.7)
	Three doses	17 (32.1)
	Four doses	33 (62.3)

Note: Percentages calculated based on respondents to the questionnaire (n = 55).

**Table 4:** Responses related to HIV care access and continuity during the COVID-19 pandemic (n = 55).

Questions	n (%)
**If you suspect you have symptoms of COVID-19, would you know who to look for medical advice?**	
Yes	43 (97.7%)
**Who would you contact if you had a fever or cough?**	
The doctor I follow	21 (38.9%)
A general practitioner	30 (55.6%)
Other	3 (5.6%)
**Have you felt any impact on the continuity of your care (e.g., withdrawal of HIV medications, routine visits, taking tests)?**	
No	40 (72.2%)
**Have you had difficulty seeing your infectious disease doctor or obtaining HIV medication due to measures taken due to the COVID-19 pandemic?**	
No, I had no difficulty getting my HIV medication	52 (94.5%)
Yes, I couldn't get my medications because they weren't available at the clinic/pharmacy	0 (0)
Yes, I didn't have money and couldn't get my meds as I had to stop working because of COVID-19 restrictions	1 (1.8%)
Yes, I couldn't go to the clinic/pharmacy due to mobility restrictions	2 (3.6%)
**During the COVID-19 pandemic, you:**	
Had a higher risk of stopping HIV medication	7 (12.7%)
Had a lower risk of stopping HIV medication	0 (0)
Didn't change your risk of stopping HIV medication	48 (87.3%)
**Did you stop your HIV treatment at any point during the COVID-19 pandemic?**	
No	52 (94.5%)

Note: Percentages calculated over the total number of participants who completed the questionnaire (n = 55).

Vaccination coverage against SARS-CoV-2 was high: 96.4% of respondents had received at least one dose, and 62.3% received four doses by the time of the study. Self-reported adherence to ART remained excellent, with 94.5% of patients reporting regular daily use of medications and only 3 (5.4%) admitting to occasional missed doses. Despite the pandemic’s social-distancing measures, 94.5% of participants maintained follow-up either in person or through remote communication (telephone, text messaging, or email).

Screening for mental-health symptoms identified 27.7% of participants with depressive symptoms (PHQ-2 ≥ 3) and 45.4% with anxiety symptoms (GAD-2 ≥ 3). Women had higher frequencies of both depression (18%) and anxiety (33%) compared with men (9% and 19%, respectively), although differences did not reach statistical significance (p > 0.05). No association was observed between mental-health symptoms and virological or immunological outcomes.

Loss to follow-up during the pandemic was low, affecting only 5.4% of patients, most of whom resumed care after relaxation of restrictive measures. No deaths were recorded among cohort participants in the study period.

## Discussion

This study examined the outpatient care of people living with HIV during the COVID-19 pandemic in a selected cohort of patients who remained engaged in follow-up at a specialized public health center in southeastern Brazil. Despite the substantial logistical challenges associated with the pandemic, virological and immunological parameters, as well as indicators of retention in care, remained stable within this cohort. These findings describe the experience of patients with continuous follow-up and available longitudinal data and should be interpreted as descriptive, without implying population-level effects or causal relationships. Together, they illustrate the capacity of this healthcare service and its patients to maintain HIV care delivery under conditions of major system disruption.

The stability of clinical indicators, evidenced by an increase in viral suppression from 77.6% to 88.7% and in CD4 counts > 200 cells/µL from 79.7% to 83.7%, suggests that ART adherence and retention in care remained effective throughout the period. Comparable findings have been reported in other Brazilian centers, where strategies such as telemedicine, remote ART prescription renewal, and partnerships with NGOs helped reduce disruptions in service delivery.^9^^,^^10^ In the studied cohort, only 5.5% of patients were temporarily lost to follow-up, far below the pre-pandemic estimates of 8% to 30% reported in Latin-American studies.^5^

Among the 33 respondents who underwent COVID-19 testing, 13 (39.4%) had positive results, and among these, 2 (15.4%) required hospitalization.^11^, ^12^, ^13^ Monteiro et al. (2022) found a 4.2% COVID-19 incidence among PLWHA in São Paulo, similar to the general population, with no deaths recorded. The clinical course was primarily linked to comorbidities rather than HIV status. These favorable outcomes were associated with high ART adherence (> 95%) and strong immune status (mean CD4+ count: 768 cells/mm³), suggesting that stable, treated HIV infection does not significantly increase the risk of severe COVID-19.^14^ The very high vaccination rate observed (96.4%) underscores the effectiveness of Brazil’s public immunization campaign and patient engagement in preventive health behaviors.

Mental-health screening revealed depressive and anxiety symptoms in 27.7% and 45.4% of participants, respectively. These proportions are lower than those documented in international studies, where the prevalence of depressive symptoms among people living with HIV during the COVID-19 pandemic ranged from 20% to 40%.^15^ These findings are in line with a recent rapid systematic review and meta-analysis, which reported a pooled prevalence of moderate-to-severe depressive and anxiety symptoms of 16.9% and 23.0%, respectively, among people living with HIV during the pandemic.^15^ The lower frequency observed in this cohort may reflect sustained psychosocial support and stable access to antiretroviral therapy, factors known to mitigate psychological distress. Nevertheless, women exhibited higher rates of both depression and anxiety, consistent with gender-related vulnerability described in prior analyses.^1^^,^^16^^,^^17^

Although an in-depth analysis of mental health determinants was beyond the scope of this study, the high prevalence of anxiety and depressive symptoms underscores the need for integrated psychological support within HIV care models, particularly during public health crises. Factors such as fear of infection, social isolation, economic instability, disruption of daily routines, and uncertainty regarding access to healthcare services may have contributed to increased psychological distress among people living with HIV.^18^ Although pre-pandemic mental health data were not available for direct comparison, similar or higher prevalence of anxiety and depression during the pandemic have been reported in PLHIV and other chronic disease populations. Importantly, these findings underscore the need for integrated mental health screening and support within HIV care services, particularly during periods of public health crisis. Given the use of brief screening instruments and the descriptive design of the study, these results should be interpreted as indicators of psychological vulnerability rather than formal psychiatric diagnoses.

Global evidence shows that the COVID-19 pandemic disrupted HIV prevention and treatment services, especially in low- and middle-income countries. According to UNAIDS, in 2020, there were 37.7 million people living with HIV worldwide, 27.5 million (73%) receiving ART, and 66% achieving viral suppression.^4^ However, several studies demonstrated that well-structured outpatient programs were able to maintain good outcomes through rapid adaptation. In Thailand, despite delayed appointments in 22% of cases, viral suppression exceeded 95%.^19^ while in Brazil, 94.5% of patients reported adequate adherence and 87.2% maintained undetectable viral load despite suspended visits.^20^

As described above, virological suppression and immunologic recovery remained high and stable during the study period, with few patients lost to follow-up within this selected cohort of individuals who maintained continuous engagement in care. Although the use of telemedicine was not systematically quantified, indirect indicators from follow-up continuity and questionnaire data suggest that approximately 30%–50% of patients may have used some form of remote communication (telephone, messaging, or email) at least once during the study period. These findings should be interpreted as descriptive observations and cannot be used to infer the effectiveness of specific adaptive measures, such as remote consultations, extended antiretroviral dispensing, or multidisciplinary coordination, which were not systematically documented at the individual level. Nevertheless, this experience illustrates how HIV care delivery can be maintained among patients retained in follow-up during periods of major health-system disruption and highlights the importance of resilient and patient-centered care models when planning responses to future public health emergencies.

This study has several limitations that should be acknowledged. First, data were collected after the acute phase of the COVID-19 pandemic and only from individuals who could be contacted and agreed to participate; therefore, patients who were lost to follow-up or did not respond may have been underrepresented, introducing selection bias and meaning that the findings, particularly those based on patient-reported data, may better reflect individuals who maintained stable engagement in care. Second, the retrospective, single-center design relies on information recorded in medical charts and on self-reported data, which are subject to recall and information bias and limit the generalizability of the findings to other settings. In addition, missing information on key demographic and clinical variables, including race, city of residence, and comorbidities, may have introduced information bias and further limit the generalizability of the findings beyond this selected cohort. Third, the absence of a comparison group precludes establishing causal relationships between the adaptive strategies implemented during the pandemic and the outcomes observed, underscoring the need for future prospective or comparative studies. Observed changes in CD4 T-cell counts or viral load may reflect the expected natural course of patients receiving stable antiretroviral therapy rather than the effect of specific interventions. Future studies incorporating comparator groups or quasi-experimental designs are needed to more robustly evaluate the effectiveness of care delivery adaptations implemented during public health emergencies. Fourth, the modest sample size and the fact that only a subset of participants completed the questionnaire may have introduced additional response bias. Fifth, laboratory monitoring during the pandemic was partially restricted, and incomplete or inconsistently recorded information in medical files may have affected the completeness and interpretation of certain clinical and laboratory variables. Sixth, although anxiety and depressive symptoms were frequently reported, an in-depth mental-health analysis was beyond the scope of this study; these findings were included primarily to contextualize their potential influence on ART adherence and healthcare engagement rather than to provide a comprehensive psychiatric evaluation. Seventh, the use of telemedicine and extended ART dispensing were emergency measure not quantitatively documented, preventing a more detailed assessment of their impact on patient outcomes. Despite these limitations, the concordance of clinical, laboratory, and behavioral observations supports the internal coherence of the findings within this selected cohort and provides a consistent descriptive picture of HIV outpatient follow-up during the study period.

## Conclusion

Despite the unprecedented challenges of the COVID-19 pandemic, outpatient care for people living with HIV in this selected cohort of patients with continuous follow-up at a specialized public health center in southeastern Brazil remained stable. Rates of adherence, viral suppression, and immunologic recovery were maintained, and loss to follow-up was minimal. These findings reflect the resilience of both patients and the healthcare service within this setting. Given the descriptive nature of the study, no causal inferences can be made regarding the effectiveness of specific adaptive measures, such as telemedicine, extended ART dispensing, or multidisciplinary coordination. Nevertheless, this experience illustrates how rapid organizational adaptations may support continuity of HIV care among patients retained in follow-up during large-scale health crises and may inform the design of sustainable care models for future emergencies.

## Ethics approval and consent to participate

The study was approved by the Research Ethics Committee of Centro Universitário FMABC (CAAE 59844222.0.0000.0082), and written informed consent was obtained from all participants in accordance with the Declaration of Helsinki and the Brazilian General Data Protection Law (LGPD, Law 13.709/2018).

## Consent for publication

All authors have read and agreed to the published version of the manuscript.

## Authors' contributions

Mariana Velho Menoia: Conceptualization; investigation; writing-original draft; data curation.

Guilherme Barbosa Pinto: Writing-original draft.

Erika Yukie Ishigaki: Formal analysis.

Nicoly Caroline de Andrade Delmondes: Data curation; investigation.

Daniel Ayabe Ninomiya: Visualization; Writing-review and editing.

Olavo Henrique Munhoz Leite: Supervision, conceptualization, review and editing.

Marcello Mihailenko Chaves Magri: Writing-original draft; Writing-review and editing; Supervision; Project administration.

All authors have approved the final manuscript draft.

## Declaration of competing interest

The authors declare no conflicts of interest.
